# Controlling the Polymorphism of Indomethacin with Poloxamer 407 in a Gas Antisolvent Crystallization Process

**DOI:** 10.1021/acsomega.2c05259

**Published:** 2022-11-18

**Authors:** Fidel Méndez Cañellas, Vivek Verma, Jacek Kujawski, Robert Geertman, Lidia Tajber, Luis Padrela

**Affiliations:** † Department of Chemical Sciences, Bernal Institute, University of Limerick, LimerickV94 T9PX, Ireland; § SSPC, the SFI Research Centre for Pharmaceuticals, Bernal Institute, University of Limerick, LimerickV94 T9PX, Ireland; ⊥ Chair and Department of Organic Chemistry, Faculty of Pharmacy, Poznan University of Medical Sciences, Grunwaldzka 6 street, Poznan60-780, Poland; ∥ Janssen Pharmaceutica NV, Beerse2340, Belgium; # School of Pharmacy and Pharmaceutical Sciences, Trinity College Dublin, College Green, Dublin 2D02 PN40, Ireland

## Abstract

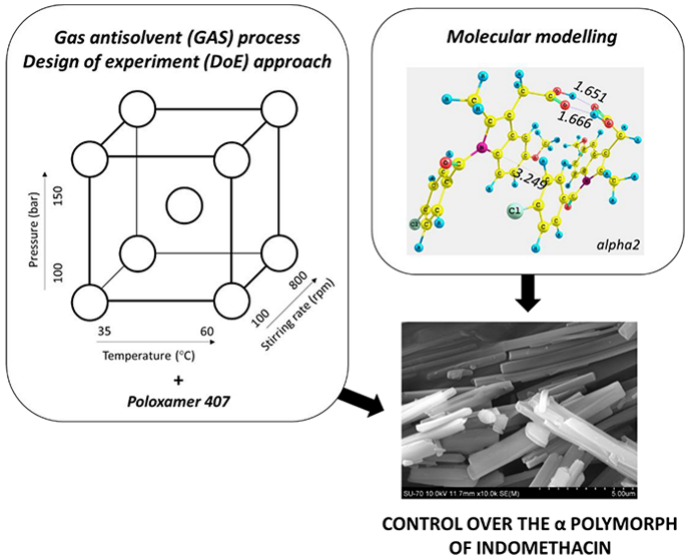

The polymorphic control of active pharmaceutical ingredients (APIs) is a major challenge in the manufacture of medicines. Crystallization methods that use supercritical carbon dioxide as an antisolvent can create unique solid forms of APIs, with a particular tendency to generate metastable polymorphic forms. In this work, the effects of processing conditions within a gas antisolvent (GAS) crystallization method, such as pressure, stirring rate, and temperature, as well as the type of solvent used and the presence of an additive, on the polymorphism of indomethacin were studied. Consistent formation of the X-ray powder diffraction-pure α polymorphic form of indomethacin by GAS was only achieved when a polymer, poloxamer 407, was used as an additive. Using the GAS method in combination with poloxamer 407 as a molecular additive enabled full control over the polymorphic form of indomethacin, regardless of the processing conditions employed, such as pressure, temperature, stirring rate, and type of solvent. A detailed molecular modeling study provided insight into the role of poloxamer 407 in the polymorphic outcome of indomethacin and concluded that it favored the formation of the α polymorph.

## 1 Introduction

Polymorphism is defined as the ability of a material to crystallize as two or more different crystal structures.^1^ The analysis of polymorphism of active pharmaceutical ingredients (APIs) is regularly conducted in the pharmaceutical industry, as each molecule has its own polymorphic landscape.^2,3^ Different crystal arrangements present different intra- and intermolecular interactions such as hydrogen bonds, van der Waals interactions, etc.^1,4^ The distinct crystalline structures of different polymorphic forms might lead to distinct physicochemical properties such as solubility, melting point, and chemical stability, which can have a significant effect on the therapeutic efficacy and bioavailability of APIs.^1,2,4,5^ The production of a specific polymorphic form often remains a challenge for the pharmaceutical industry due to the complex polymorphic landscape chemical entities.^1,4,6^ Other challenges include solid form conversion during storage, packaging, or processing of the product.^1^ Crystallization methods need to be robust and accurately designed to enable controlled formation of the desired polymorphic form.^3^ The method selected needs to be reproducible, and a specific polymorph must be consistently obtained while no unexpected conversion into other polymorphs or solid forms (e.g., solvates, amorphous, salts, hydrates) should occur.^1,5^ However, small-scale crystallization events can be challenging to control, as nucleation may present a strong nonlinear behavior with high fluctuations in supersaturation.^7^ Consequently, it is expected that in small scale experiments the polymorphic form is difficult to control (if there are multiple polymorphic forms).

Several crystallization techniques and strategies have been reported in the literature to control the polymorphism of APIs. Some of the most common techniques include antisolvent crystallization, cooling crystallization, and solvent evaporation.^4^ The resultant polymorph may vary depending on the solvent composition, the presence of additives, and the processing conditions chosen, which include the temperature of crystallization, saturation levels, and agitation.^2,4^ The presence of additives can influence the nucleation and growth kinetics of the APIs, and hence can provide a major influence in controlling the formation or inhibition of a particular polymorph.^8,9^ For instance, Renuka and Srinivasan studied the effect of the additive sodium nitrate in the nucleation of two polymorphs of glycine.^10^ They observed that the concentration of sodium nitrate controlled the formation of a monomer or a dimer of glycine that determined the polymorphic outcome.^10^ Fine-tuning additive selection and crystallization processing conditions to achieve control over the crystal morphology, size, surface area, and physicochemical properties of APIs.^2,11^

Crystallization methods based on the antisolvent role of supercritical CO_2_ tend to promote the formation of metastable polymorphic forms of APIs, and have also been reported to generate polymorphic forms that other techniques are not able to reproduce.^12−14^ Supercritical CO_2_ antisolvent techniques present several attractive characteristics which include the use of mild processing temperatures, easily tunable processing conditions, no risk of forming hydrates (contrarily to liquid antisolvent methods which use water as the antisolvent), and allow the formation of solvent-free dried products, as the remaining organic solvent(s) is/are removed from the final product during flushing with CO_2_ after the crystallization process is completed.^15−18^ Specifically, the gas antisolvent (GAS) crystallization method has been reported in the literature for the production of micron and nanosized particles of APIs.^18−23^ Contrarily to other techniques that also use supercritical CO_2_ as an antisolvent (e.g., supercritical antisolvent crystallization (SAS), expanded liquid antisolvent (ELAS), atomization and antisolvent crystallization (AAS)), GAS does not involve an atomization step.^15^ Furthermore, the polymorphism of APIs is challenging to control using the GAS method as during the experimental process, the crystallization occurs in a transitory regime since both the pressure and concentration vary. For other supercritical antisolvent methods such as the SAS process, the crystallization occurs in a permanent regime as the pressure, temperature, and concentration remain constant throughout the production of particles.^15,24,25^ For that reason, the powders formed by GAS process may exhibit inhomogeneous characteristics in regards to particle size distribution^23^ and polymorphic nature.^19^ A strategy to overcome this limitation is the use of additives which can promote further control over the polymorphic form and crystal morphology of the API particles obtained.^26,27^ Long et al. used the GAS method and a design of experiments approach (DoE) to achieve control over the polymorphic form II and III of carbamazepine using sodium stearate and sodium dodecyl sulfate as additives, respectively.^19^

Indomethacin is an acidic nonsteroidal anti-inflammatory drug (NSAID) that presents analgesic, anti-inflammatory, and antipyretic properties,^28^ and indomethacin particles have been prepared with techniques based on supercritical CO_2_ such as SAS,^29,30^ AAS,^29^ GAS,^31^ and others.^32^ This API presents eight known polymorphic forms. Surwase et al. reported the α, γ, ε, δ, ζ, and η forms, the β form has been reported by Kaneniwa et al. and Lin, and the τ form has been reported by Van Duong et al.^33−36^ Among all the polymorphs, the γ polymorph is the thermodynamically stable form and the α polymorph is considered a metastable form.^35^ Yoshioka et al. reported that indomethacin is a monotropic system between 30 and 60 °C with the γ form being the most stable form.^37^ Nevertheless, the α form has been reported to gradually transform to the γ form depending on the heating rate and environmental conditions.^34,37^ Both polymorphic forms of indomethacin are desirable for pharmaceutical formulations despite their different physicochemical characteristics. For instance, the stable form (γ polymorph) is less likely to undergo solid-state transformations into other polymorphic forms, and the metastable form (α polymorph) presents enhanced apparent solubility.^37^ Moreover, the α polymorph presents improved tabletability compared to the γ polymorph.^64^

Van Duong et al. used various additives, including poloxamer 407, to study the polymorphism of indomethacin by melt crystallization.^36^ The newly discovered τ form and the α polymorph of indomethacin were formed in the presence poloxamer 407, and thus, this polymer attracted our attention as a potential additive to control the polymorphism of this API. Poloxamer 407 is included in the Food and Drug Administration (FDA) Inactive Ingredients Database, and it is widely used in oral, ophthalmic and topical formulations and regarded as nontoxic and nonirritant material.^38,39^ Poloxamers are used as emulsifying agents, stabilizing agents and/or as tablet additives at concentrations up to 10%.^38^ While no literature report on the control of the polymorphism of indomethacin using poloxamer 407 with a CO_2_-based technique has been published to date, other studies have shown the production of indomethacin particles using top-down techniques using this additive. Kuroiwa et al. and Malamatari et al. used poloxamers to produce stable suspensions of indomethacin using wet-milling.^40,41^ Kuroiwa et al. used poloxamer 407 to produce stable (at 25 °C for 1 week) nanosuspensions of the α and γ polymorphs.^40^ They observed, with suspended-state ^13^C pulse saturation transfer (PST)/magic-angle spinning (MAS) nuclear magnetic resonance (NMR) measurements, that the polyphenylene oxide chain of poloxamer 407 is weakly associated with the surface of indomethacin particles via hydrophobic interactions.^40^

In this work, the GAS process is used to crystallize distinct polymorphic forms of indomethacin (i.e., α and γ polymorphs). The influence of GAS processing variables such as temperature, agitation rate, pressure, and the presence of additive poloxamer 407 on the polymorphic form of indomethacin form was assessed. Furthermore, a detailed analysis of the experimental results was conducted using molecular modeling to gain insight on how the additive selected (poloxamer 407) governed the polymorphic outcome of indomethacin. To the best of our knowledge, intermolecular interactions between indomethacin molecules and poloxamer 407 have not been investigated in the literature to date. This is the first report analyzing the influence of poloxamer 407 as an additive on the polymorphic form of indomethacin in CO_2_-based particle production technique.

## 2 Materials and Methods

### 2.1 Materials

Indomethacin (γ polymorph) was purchased from Baoji Guokang Bio-Technology Co. Ltd. (China). Poloxamer 407 (Kolliphor P407) was sourced from BASF (Germany). The solvents used were acetone (≥99.8%) and ethyl acetate (HPLC grade) obtained from Fisher Chemicals (Ireland). Carbon dioxide (99.98%) was supplied by BOC (Ireland).

### 2.2 Methods

#### 2.2.1 Sample Preparation

In the gas antisolvent (GAS) experiments, acetone and ethyl acetate were used as solvents, as indomethacin is soluble in both solvents.^42,43^ The solubilities of indomethacin in acetone and ethyl acetate at 25 °C are 113 and 41 mg/mL, respectively.^42,43^ The solubilities were measured by Takebayashi et al. by a statistic analytical method and were determined with UV–vis absorbance.^43^ In each experiment, 10 mg of indomethacin were dissolved in 0.5 mL of solvent inside a 1.5 mL Eppendorf tube using ultrasonic treatment for 5 min and moderate manual shaking. When indicated in the following sections (Section 3.3), 2.5 mg of poloxamer 407 was added to the indomethacin solution. The ratio of indomethacin to poloxamer 407 was set to 4:1 w/w based on a preliminary screening and Duong et al.^36^ The solutions were then filtered through a Sartorius 0.20 μm syringe filter and a 2 mL BD Discardit II syringe to remove any undissolved material.

#### 2.2.2 Gas Antisolvent Crystallization (GAS)

**Figure 1:**
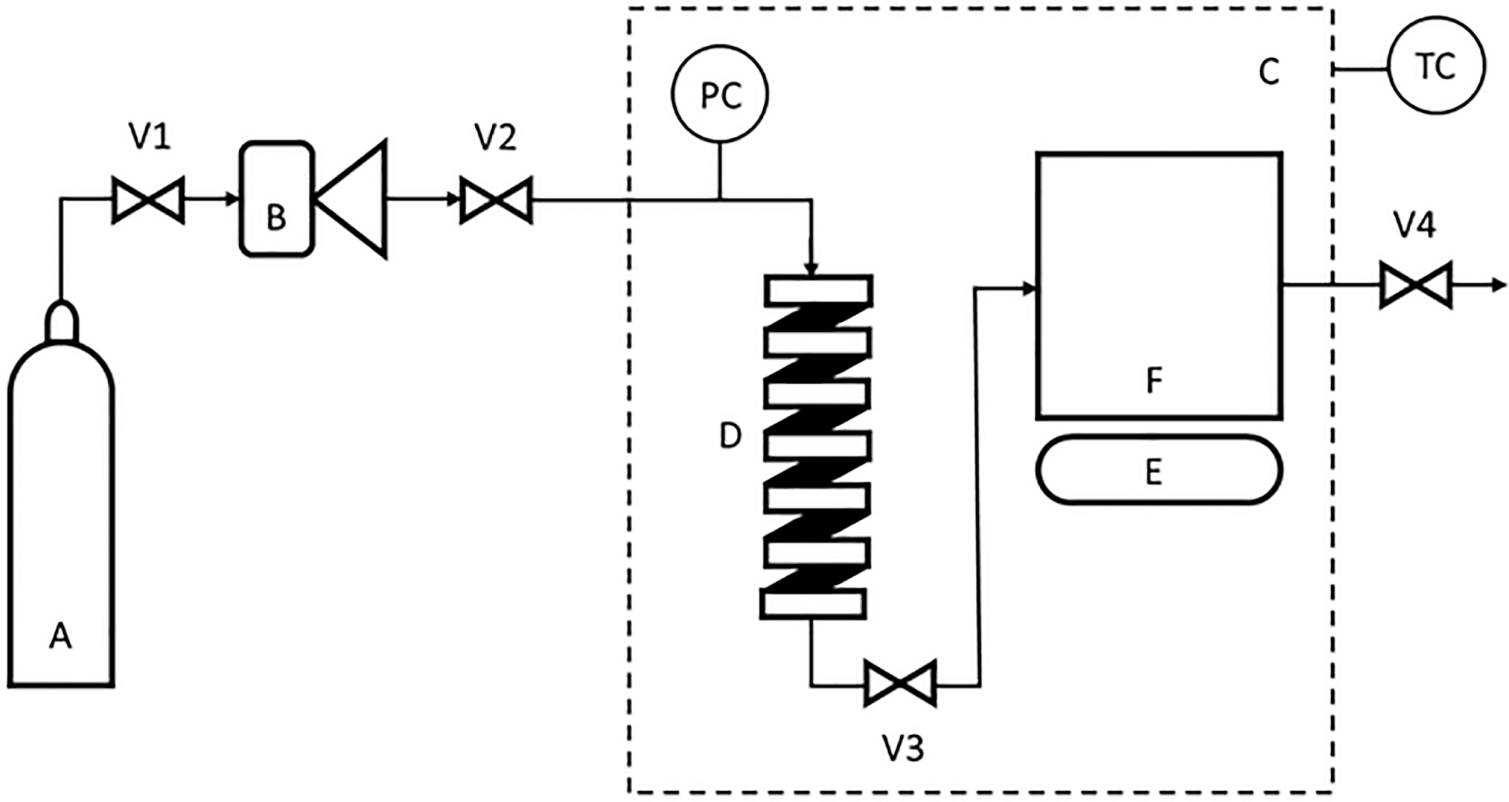
Schematic diagram of the gas antisolvent (GAS) apparatus. (A) CO_2_ cylinder, (B) cooler and gas compressor; (C) temperature-controlled (TC) air chamber; (D) high-pressure storage coil; (E) magnetic stirrer; (F) high-pressure vessel; V1, 2, 3, 4, valves; PC: pressure controlled.

Figure 1 presents a schematic diagram of a custom-built gas antisolvent (GAS) process. It consists of a 15 cm^3^ high-pressure stainless steel storage coil (D in Figure 1) and a 10 cm^3^ stainless steel high-pressure vessel (F in Figure 1) where the crystallization/precipitation process took place. The temperature and pressure (Table 1) of the high-pressure vessel was monitored using a T-type thermocouple and a pressure transducer (Omega model PX603). The temperature of the high-pressure vessel and storage coil was monitored with a temperature-controlled air chamber (C in Figure 1). A borosilicate window in the high-pressure vessel allowed the visualization of the precipitation process during the experiments. The maximum pressure that the borosilicate window can withstand is 20.0 MPa and this is the limiting factor for the design of experiments approach (Section 2.2.3). A Teledyne ISCO 260D pump (B in Figure 1) was used to load the CO_2_ (A in Figure 1) into the storage coil before being introduced into the high-pressure vessel. A solution containing 10 mg of indomethacin dissolved in 0.5 mL of acetone/ethyl acetate with/without poloxamer 407 was placed inside the high-pressure vessel and compressed with CO_2_ up to the desired pressure and temperature until crystallization occurred. During the addition of CO_2_, the solution was magnetically stirred (with a bar of 6 mm × 3 mm) to improve the mixing with the CO_2_. After 5 min, magnetic stirring was turned off, and the valve V4 in Figure 1 was opened to continuously flush supercritical CO_2_ and organic solvent through the high-pressure vessel out to the vent. The CO_2_ was flushed through the high-pressure vessel for 30 min. After flushing was completed, the vessel was depressurized and the resulting material was collected and stored in airtight containers for further characterization. The GAS setup used in this work is that as used by Long et al.^19^

**Table 1:** Experimental Variables of the GAS Process (Pressure, Temperature, and Stirring Rate), Solvents (Acetone (Ac) and Ethyl Acetate (Et)), and Additive (Poloxamer 407 (P)) Explored in This Work^a^

DoE point	additive	solvent	pressure (MPa)	*T* (°C)	stirring rate (RPM)	solid form obtained
IndAc 1	no additive	acetone	10.0	35	100	γ/solvate
IndAc 2			15.0	35	100	α/solvate
IndAc 3			15.0	35	800	γ/α + γ
IndAc 4			10.0	35	800	γ/α + γ
IndAc 5			12.5	48	450	γ/α + γ
IndAc 6			10.0	60	100	α
IndAc 7			15.0	60	100	α
IndAc 8			15.0	60	800	α
IndAc 9			10.0	60	800	α/α + γ
IndEt 10		ethyl acetate	10.0	35	100	α
IndEt 11			15.0	35	100	α + γ
IndEt 12			15.0	35	800	γ
IndEt 13			10.0	35	800	γ/α + γ
IndEt 14			12.5	48	450	α/α + γ
IndEt 15			10.0	60	100	α
IndEt 16			15.0	60	100	α
IndEt 17			15.0	60	800	α/α + γ
IndEt 18			10.0	60	800	α/γ
IndAcP 19	poloxamer 407	acetone	10.0	35	100	α
IndAcP 20			15.0	35	100	α
IndAcP 21			15.0	35	800	α
IndAcP 22			10.0	35	800	α
IndAcP 23			12.5	48	450	α
IndAcP 24			10.0	60	100	α
IndAcP 25			15.0	60	100	α
IndAcP 26			15.0	60	800	α
IndAcP 27			10.0	60	800	α
IndEtP 28		ethyl acetate	10.0	35	100	α
IndEtP 29			15.0	35	100	α
IndEtP 30			15.0	35	800	α
IndEtP 31			10.0	35	800	α
IndEtP 32			12.5	48	450	α
IndEtP 33			10.0	60	100	α
IndEtP 34			15.0	60	100	α
IndEtP 35			15.0	60	800	α
IndEtP 36			10.0	60	800	α

a The resulting solid-state form(s) are also listed. α, α polymorph of indomethacin; γ, γ polymorph of indomethacin; α + γ, mixture of the α and γ polymorphs of indomethacin; solvate, acetone solvate of indomethacin.

#### 2.2.3 Design of Experiments (DoE) Approach

Gas antisolvent processing variables such as pressure, temperature, and agitation rate may potentially affect the solid-state, particle size, and morphology of API particles produced.^18,21^ This study used a three-factor, two-level DoE with two additional points to study the influence of the pressure, the temperature of the high-pressure vessel and stirring rate. The minimum and maximum values for the variables were selected according to the pressure limit (20 MPa) of the GAS equipment used and the temperature requirement to have CO_2_ in the supercritical state, as established for Long et al.^19^ Additionally, the effect of two different solvents, acetone and ethyl acetate, and of the presence of the additive poloxamer 407 were also explored. Table 1 lists the variables selected for all DoE points and the solid-state outcome obtained for the indomethacin samples produced by the GAS process. Each of the DoE experiments were performed in duplicate. A schematic representation of the DoE is provided in Figure S1.

The categorical outcome (i.e., solid form) results could not be quantitatively analyzed. If not properly calibrated XRPD is not a quantitative method, and hence, XRPD was not considered for quantitative analysis. Consequently, the focus was not on a numerical model and the general trends were observed.

The nomenclature established for the samples is abbreviated as indomethacin (Ind), ethyl acetate (Et), acetone (Ac), and poloxamer 407 (P). For instance, an indomethacin sample produced using acetone as a solvent, and poloxamer 407 as an additive was abbreviated as IndAcP.

#### 2.2.4 X-ray Powder Diffraction (XRPD)

XRPD in reflection mode was performed at ambient conditions using an X’ Pert PRO MPD XRPD (PANalytical, Philips) and an Empyrean diffractometer (PANalytical, Philips), both equipped with Cu-α radiation (λ = 1.5406 Å) at a voltage of 45 kV, and a current of 40 mA. The instruments were operated in the continuous scan mode and the samples were analyzed in the angular range 4–35° (2θ) with a step size of 0.013° (2θ) and a measuring time per step of 18.87 s.

#### 2.2.5 Scanning Electron Microscopy (SEM)

Scanning electron microscopy was performed using a SU70 Hitachi (Hitachi Inc., Japan) scanning electron microscope instrument. Samples were mounted onto 15 mm aluminum stubs with carbon tabs. The samples were coated by an ultrathin gold layer prior to analysis using an Emitech K550 (Emitech, UK) sputter coater at 20 mA for 45 s.

#### 2.2.6 Differential Scanning Calorimetry (DSC)

Thermal analysis (by DSC) was performed using a Netzsch Polyma 214 DSC (Netzsch, Germany) that was calibrated using Sn (Tin) as a standard. Samples (5 to 10 mg) were crimped in nonhermetic aluminum pans (25 μL) and scanned at a heating rate of 10 °C/min from 25 to 200 °C (above the melting temperature of indomethacin) under a nitrogen purge. The instrument was equipped with a refrigerated cooling system.

#### 2.2.7 Molecular Modeling

The quantum mechanics (QM) computations were carried out using the Gaussian 16 C.01 program.^44^ The density functional theory (DFT) formalism^45^ with the B97D3 functional^46^ was used in the gaseous phase. The crystal structures of the α and γ polymorphic forms of indomethacin were obtained from Cambridge Structural Database (CSD) (INDMET02 and INDMET03). Dimers with different configurations were optimized using the 6-31G(d,p) basis set using very tight criteria of optimization. The interaction energy was estimated using the B97D3/6-311++G(d,p) level of theory with the counterpoise corrected method and basis set superposition error (BSSE)^47,48^ as well as symmetry-adapted perturbation theory (SAPT) analysis, the SAPT0 approach. Psi4 1.3.2 software^49^ was used to treat the dimers as a closed-shell system,^50,51^ and the recommended jun-cc-pVDZ basis set was utilized.^52^

MOPAC2016 software^53^ and the PM7 method^54^ were used for semiempirical calculations. For the interaction enthalpy calculations (based on the heat of formation values), a previous protocol was used.^55,56^ The poloxamer 407 monomer was constructed manually. The genetic algorithm (GA) method implemented in the AutoDock Vina program^57^ was employed to provide the appropriate binding orientations and conformations of the compounds in the presence of the polymer. The geometries of indomethacin molecules taken from the previously optimized “alpha2” and “gamma2” dimers (using the DFT formalism) were considered as ligands. Polar hydrogen atoms were added, partial charges were assigned to the poloxamer monomer, then the residues were saturated with hydrogen atoms. A grid box (center _x = 116.708 Å, center_y = 125.391 Å, center_z = 123.507 Å, size_x = 162 Å, size_y = 162 Å, size_z = 162 Å) was defined to carry out the docking simulation. The outputs (*.pdbqt files) after the docking procedure were used for further molecular dynamics (MD) investigations.

For the MD calculations, GROMACS 2016.4^58,59^ was employed to simulate the complexes. The Amber99SB-ILDN force field^60^ was used to parametrize the atoms in poloxamer. The general GAFF force field^61^ was utilized to represent the ligands and their topology was defined with the help of Topolbuild 1.2.1.^59^ Finally, the complexes were inserted into the cubic boxes (10 × 10 × 10 nm). The complex consisted of one molecule of the poloxamer monomer and one ligand. The complexes were first minimized using the steepest descent scheme. Then, the minimized configurations were relaxed in NVT and NPT ensembles with 500 ps MD length per simulation. The complexes were restrained by NVT simulations using a small harmonic force. For the complexes free of restraints, NPT MD simulations were adopted. The relaxed system was then used as an initial conformation for 20 ns MD simulations. The time step used throughout the MD calculations was 2 fs. Chemcraft 1.7 software was utilized for visualization of all optimized systems.^62^

## 3 Results and Discussion

### 3.1 Crystallization of Indomethacin by Gas Antisolvent (GAS) without Poloxamer 407

**Figure 2:**
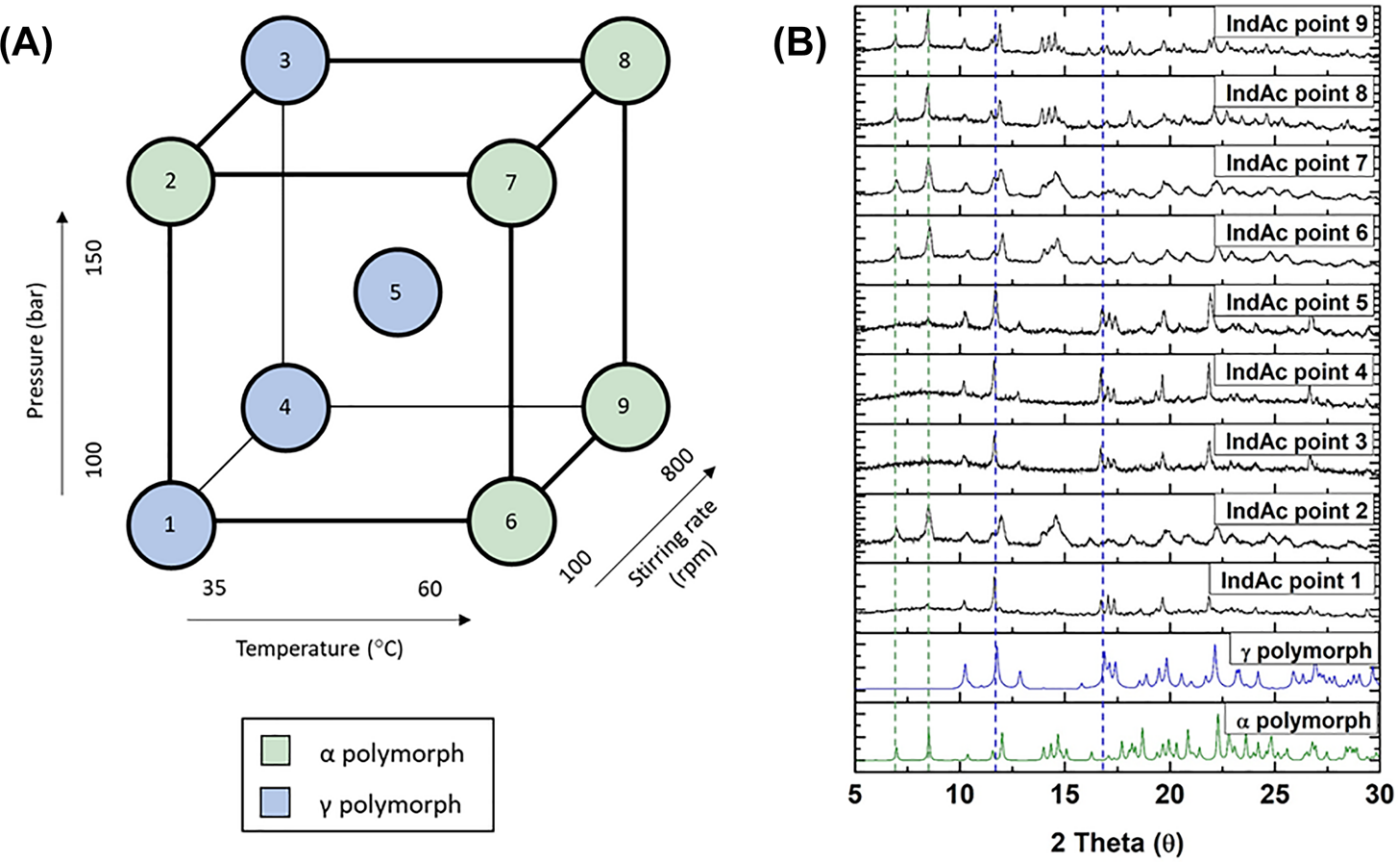
(A) Design of experiments (DoE) schematic to investigate the impact of the pressure, the temperature, and stirring the rate as the process variables on the polymorphic outcome of indomethacin particles produced by the gas antisolvent (GAS) process, using acetone as the solvent. (B) X-ray powder diffraction (XRPD) patterns of the α and γ polymorphs of indomethacin from the Cambridge Structural Database (CSD) and DoE samples produced by the GAS method. Experimental conditions as described in Table 1 (DoE points IndAc 1–9). Green dotted lines indicate the characteristic peaks of the α polymorph at 7° and 8.5° 2θ, while the blue dotted lines indicate the characteristic peaks of the γ polymorph at 11.8° and 17° 2θ.

**Figure 3:**
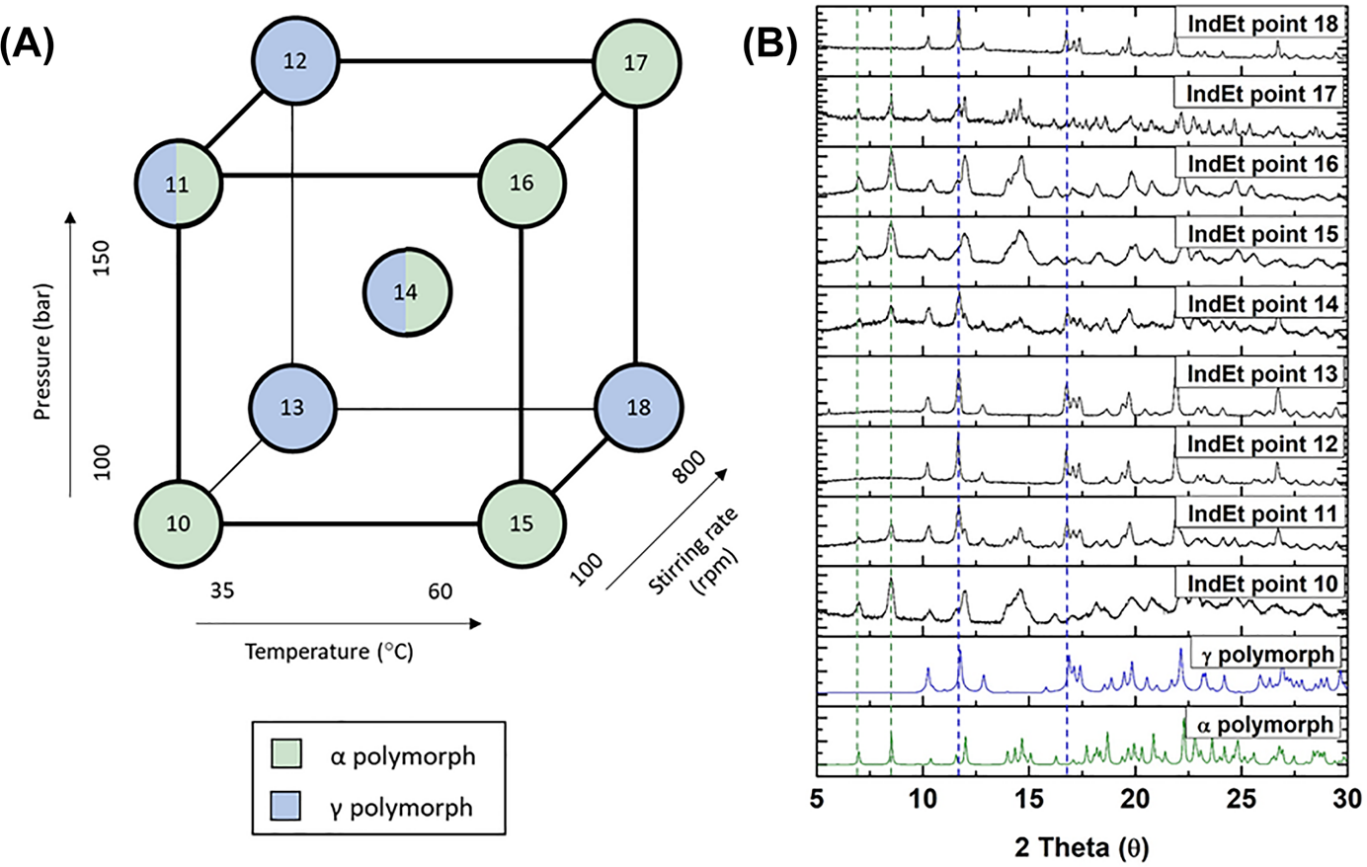
(A) Design of experiments (DoE) schematic to investigate the impact of the pressure, the temperature, and the stirring rate as the process variables on the polymorphic outcome of indomethacin particles produced by the gas antisolvent (GAS) process, using ethyl acetate as the solvent. (B) X-ray powder diffraction (XRPD) patterns of the α and γ polymorphs of indomethacin from the Cambridge Structural Database (CSD) and DoE samples produced by the GAS method. Experimental conditions as described in Table 1 (DoE points IndEt 10–18). Green dotted lines indicate the characteristic peaks of the α polymorph at 7° and 8.5° 2θ, while the blue dotted lines indicate the characteristic peaks of the γ polymorph at 11.8° and 17° 2θ.

Figures 2 and 3 illustrate the X-ray powder diffraction (XRPD) patterns of indomethacin samples obtained from the GAS process, using acetone or ethyl acetate as solvents. The duplicates are presented in Figures S2 and S3. The γ polymorph of indomethacin presents characteristic peaks at 10.2°, 11.8°, 17.0°, and 19.9° 2θ, while the α polymorph presents characteristic peaks at 7.0°, 8.5°, 11.6°, 12.0°, and 14.0° 2θ.^35^ In the XRPD patterns presented in Figures 2 and 3, it can be observed that the singlets at 7.0° and 8.5° 2θ correspond to the α polymorph, and that the singlet at 11.8° 2θ and the triplet at 17.0° 2θ correspond to the γ polymorph. The XRPD patterns were mainly compared with the Cambridge Structural Database (CSD) patterns INDMET02 (α polymorph) and INDMET03 (γ polymorph). Nevertheless, there were XRPD patterns in the duplicate experiments (Figure S2, IndAc experiments 1, 2) that did not fit to any of the reported indomethacin polymorphic forms from the CSD but instead matched an indomethacin acetone solvate pattern reported by Malwade and Qu.^42^ The acetone solvates produced in our work were generated at a lower temperature (35 °C) and lower stirring rate (100 rpm) by the GAS method. It could be hypothesized that at the previous conditions (35 °C and 100 rpm) the critical activity of acetone for the solvate formation could be exceeded.

It was observed that the only reproducible experiments (i.e., both duplicates presented the same polymorph outcome) were IndAc experiments 6, 7, and, 8. When ethyl acetate was used as solvent, as presented in Figure 3, IndEt experiments 13, 14, 17 and, 18 were the only points that were not reproducible. The scale of the experiments conducted might have affected the reproducibility of the experiments, as the volume of solvent where the API was dissolved was 0.5 mL. Therefore, spontaneous nucleation at that scale is challenging to reproduce and might tend to randomize polymorphic outcomes. Nonetheless, for the IndAc and IndEt experiments (Table 1) there was a predominance of the α polymorph of indomethacin at 60 °C independently of the stirring rate and pressure used. For the IndEt experiments, the prevalence of the α polymorph at 60 °C was inferior compared to the IndAc experiments but still notable. Contrarily, the γ polymorph and a mixture of both forms were more predominant at 35 °C. This fact is in agreement with the literature which states that the formation of the α polymorph of indomethacin is favored at temperatures above the glass transition temperature (*T*_g_), while the formation of the γ polymorph is favored at temperatures below *T*_g_.^35,63,64^ Since the *T*_g_ of indomethacin has been reported to be within the range of 42–45 °C, it could be expected that at 60 °C the formation of the α polymorph is favored, while at 35 °C, the γ polymorph is more predominant.^63,65^

Taking into account the experiments conducted, the influence of the other processing variables (stirring rate, pressure and solvent used) was observed to have a less effect than the temperature. Apart from the processing conditions discussed, the mole fractions of CO_2_ and indomethacin were analyzed in Table S1, which is presented in the Supporting Information.

In this section, the lack of consistency in the polymorphic outcome within the duplicate experiments underscores the stochastic nature of the nucleation events, particularly at this small scale. Despite observing some correlation between the temperature and the polymorphic outcome in the DoE (as reported in the literature), the small-scale crystallisation event proves challenging to control. A larger pool of experiments would be required to correlate the other experimental variables (pressure, stirring rate, type of solvent used) with the polymorphic outcome.

**Figure 4:**
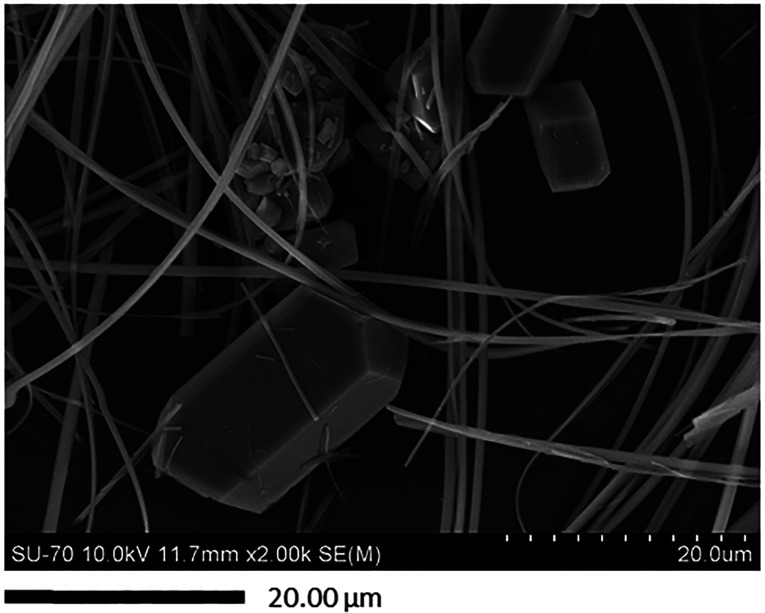
Scanning electron microscopy (SEM) image of indomethacin particles produced by the gas antisolvent (GAS) process for the design of experiments (DoE) point IndEt 11.

The morphology of the particles was further explored using scanning electron microscopy (SEM). For instance, Figure 4 presents an image of the indomethacin sample produced from IndEt point 11 where in both duplicates, a mixture of the α and γ polymorphs was obtained. It can be observed that two distinct particle morphologies corresponding to the α and γ polymorphs was obtained. As reported in the literature, the α polymorph of indomethacin presents an acicular or needlelike shape, and the γ polymorph has a platelike shape.^30,42,66^

### 3.2 Crystallization of Indomethacin by Gas Antisolvent (GAS) with Poloxamer 407

**Figure 5:**
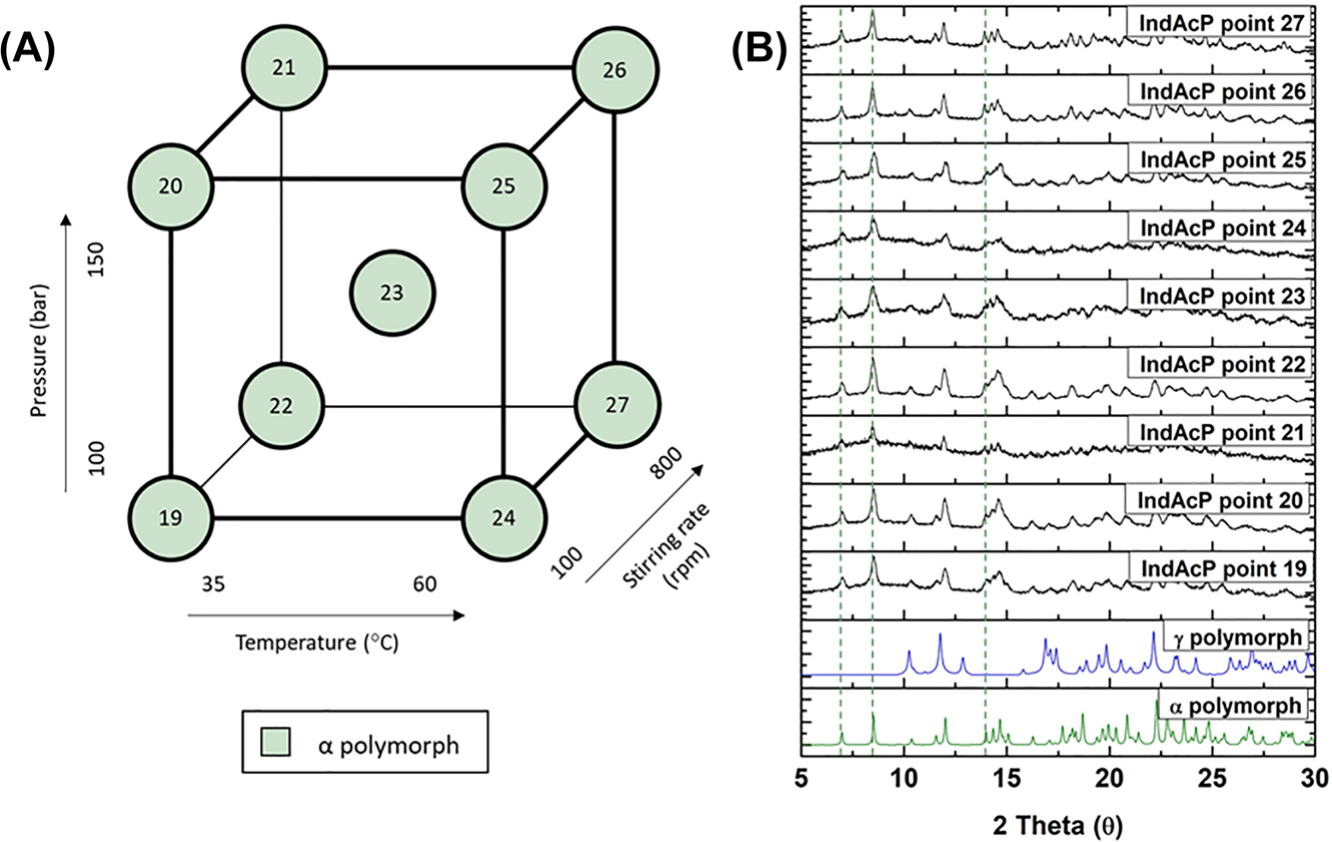
(A) Design of experiments (DoE) schematic to investigate the impact of the pressure, the temperature, and the stirring rate as the process variables on the polymorphic outcome of indomethacin particles produced by the gas antisolvent (GAS) process, using acetone as the solvent and poloxamer 407 as the additive. (B) X-ray powder diffraction (XRPD) patterns of the α and γ polymorphs of indomethacin from the Cambridge Structural Database (CSD) and DoE samples produced by the GAS method. Experimental conditions as described in Table 1 (DoE points IndAcP 19–27). Green dotted lines indicate the characteristic peaks of the α polymorph at 7°, 8.5°, and 14.0° 2θ.

**Figure 6:**
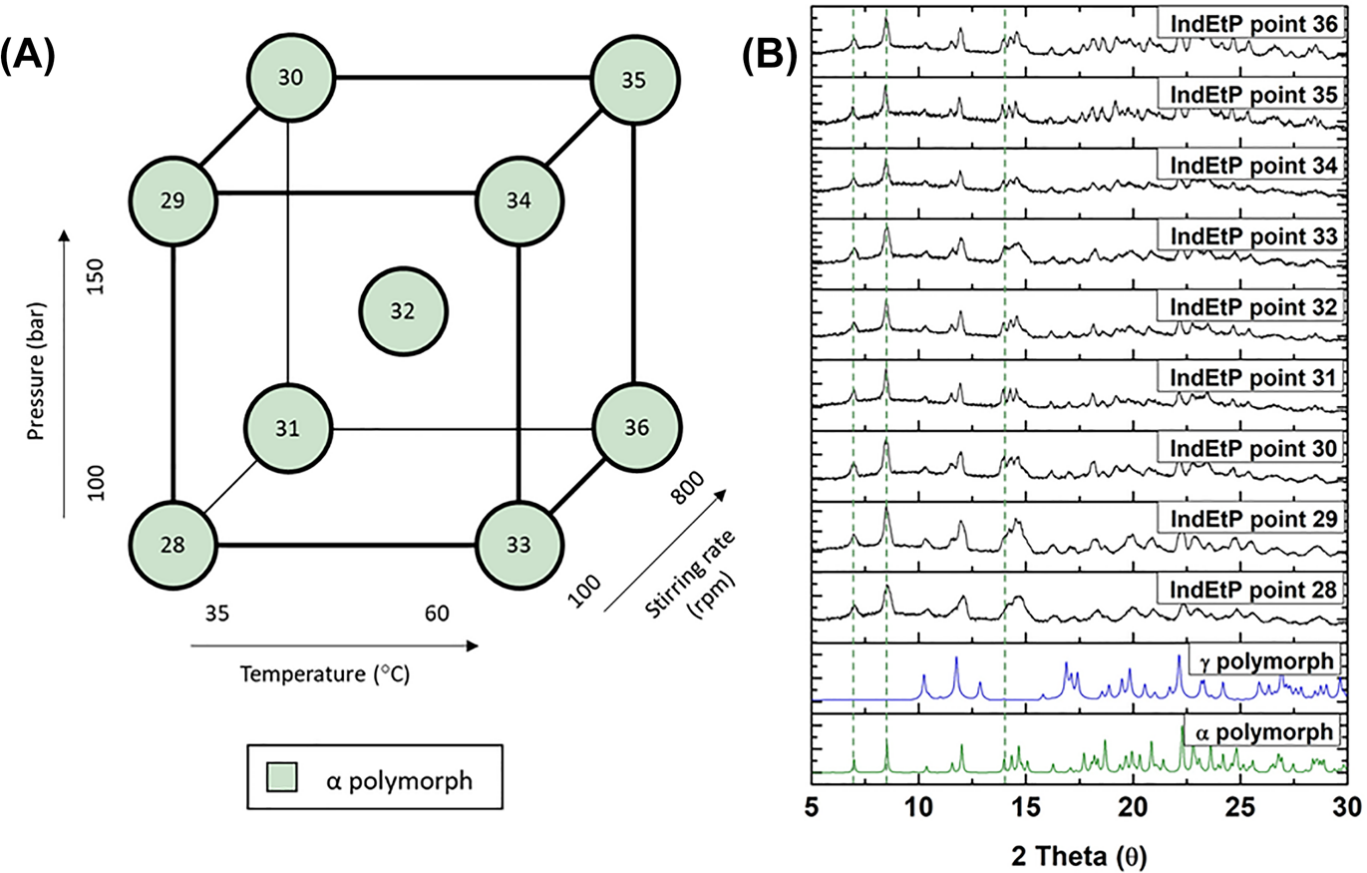
(A) Design of experiments (DoE) schematic to investigate the impact of the pressure, the temperature, and the stirring rate as the process variables on the polymorphic outcome of indomethacin particles produced by the gas antisolvent (GAS) process, using ethyl acetate as the solvent and poloxamer 407 as the additive. (B) X-ray powder diffraction (XRPD) patterns of the α and γ polymorphs of indomethacin from the Cambridge Structural Database (CSD) and DoE samples produced by the GAS method. Experimental conditions as described in Table 1 (DoE points IndEtP 28–36). Green dotted lines indicate the characteristic peaks of the α polymorph at 7°, 8.5°, and 14.0° 2θ.

The effect of the additive, poloxamer 407, on the polymorphism of indomethacin using the GAS process was studied. A 4:1 w/w ratio of indomethacin to poloxamer 407 was used in each experimental run (for DoE points 19 to 36). It was observed that the α polymorph of indomethacin was obtained for all the DoE points when using poloxamer 407. In Figures 5 and 6, it is clearly observed that poloxamer 407 promotes the formation of the α polymorph of indomethacin, irrespective of the processing conditions used for pressure, temperature, stirring rate, and type of solvent.

By comparing the two sets of experiments presented in Sections 3.1. and 3.2, it can be concluded that the stochastic nucleation behavior seen for the samples described in Section 3.1. is no longer observed for the samples coprocessed with the additive (described in this section). Therefore, polymorphic control is achieved when poloxamer 407 is used in the experiments.

**Figure 7:**
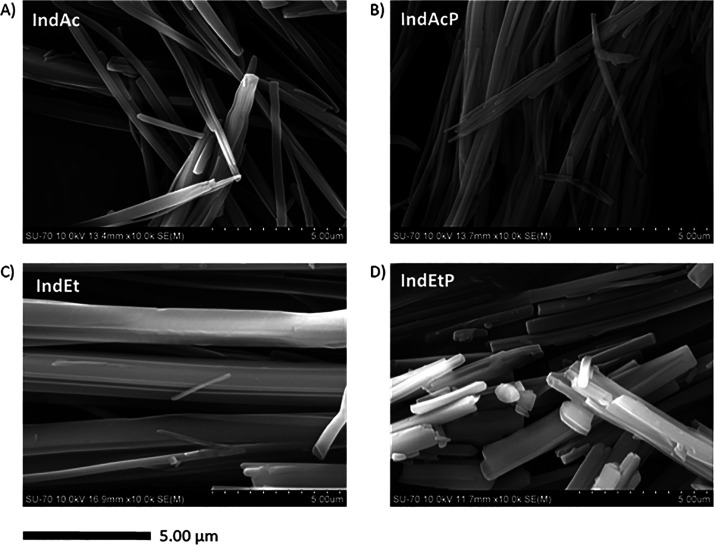
Scanning electron microscopy (SEM) images of indomethacin samples produced from different GAS experiments, corresponding to DoE points (A) 6, (B) 24, (C) 15, and (D) 33. The temperature for all the experiments was 60 °C, the pressure was 10 MPa, and the stirring rate of the solution was 100 rpm. (A) IndAc: Acetone used as the solvent. (B) IndAcP: Acetone used as the solvent and poloxamer 407 as the additive. (C) IndEt: Ethyl acetate used as the solvent. (D) IndEtP: Ethyl acetate used as the solvent and poloxamer 407 as the additive.

Moreover, the effect of the solvent used and the addition of poloxamer 407 on the morphology of indomethacin were also studied. Figure 7 compares different SEM images of indomethacin samples produced from DoE points 6 (no additive was used; the solvent used was acetone), 15 (no additive was used; the solvent used was ethyl acetate), 24 (poloxamer 407 was used as additive; the solvent used was acetone), and 33 (poloxamer 407 was used as the additive; the solvent used was ethyl acetate), where the α form of indomethacin was consistently obtained in all cases. These DoE points were conducted at the same process conditions of temperature (60 °C), pressure (10.0 MPa), and stirring rate (100 rpm). No significant differences in particle shape were observed due to the presence of poloxamer 407 or from the different solvents used (acetone and ethyl acetate). Due to the particle shape presented, the particle size could not be measured accurately, as the width of the needles was in the submicrometer range while the length was tens of micrometers.

Macroscopically, cotton-like agglomerates were observed in the samples where the α polymorph was obtained. This observation is in agreement with that obtained by Wada et al., where α polymorph of indomethacin was produced using a liquid antisolvent crystallization method, with an electrolyte aqueous solution as the antisolvent.^67^

**Figure 8:**
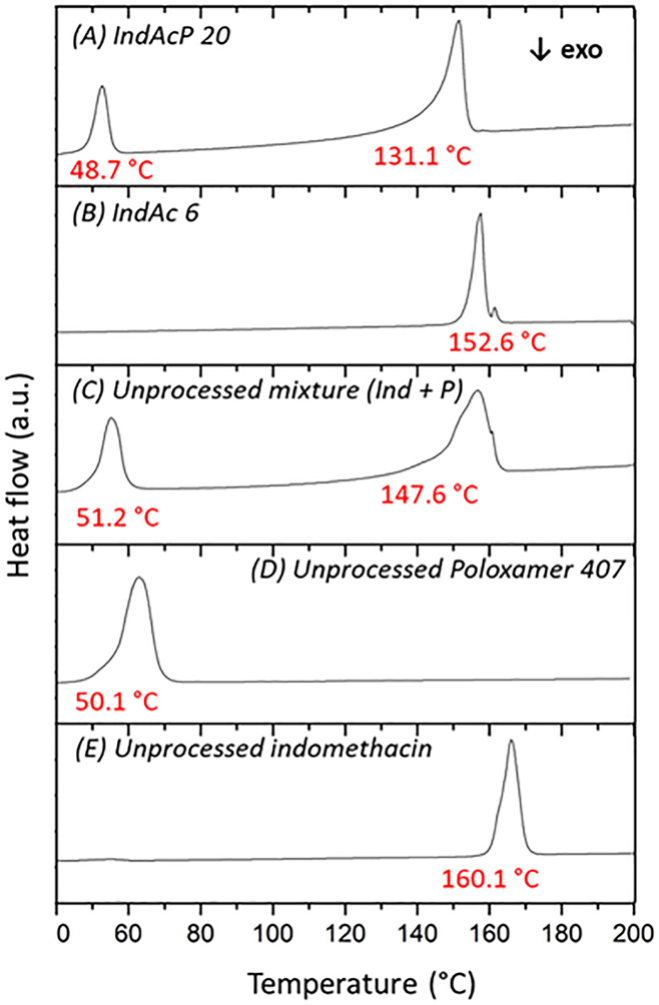
Differential scanning calorimetry (DSC) analysis of: (A) DoE point IndAcP 20, (B) DoE point IndAc 6, (C) physical mixture of unprocessed indomethacin (γ polymorph) and unprocessed poloxamer 407, (D) unprocessed poloxamer 407, (E) unprocessed indomethacin (γ polymorph). The onset temperatures are presented in red at the bottom of each peak. They represent the intersection point of the extrapolated baseline and the inflectional tangent at the beginning of the melting peak. The scans were performed at 10 °C/min. Ind: Indomethacin. P: Poloxamer 407.

The influence of poloxamer 407 on the thermal properties of the particles collected was studied by DSC, as presented in Figure 8. The reported onset melting temperatures for indomethacin are 149–154 °C and 158–161 °C for the α and γ polymorphs, respectively.^42,68,69^

The unprocessed indomethacin (γ polymorph) and the experimentally obtained α polymorph of indomethacin (as confirmed by XRPD) produced using the GAS method (IndAc 6) presented onset temperatures of 160.1 and 152.6 °C, respectively, which is in agreement with the reported literature. Regarding the α polymorph, DSC revealed a double peak of the α polymorph that suggests recrystallization of the α polymorph to the γ polymorph (previously reported in the literature).^37,70^ The presence of poloxamer 407 significantly depressed the melting point of indomethacin (γ polymorph) in the physical mixture (Figure 8C) comprising equivalent quantities of the API and polymer as those in the coprocessed sample, shifting the melting onset by nearly 13°. In the GAS sample containing the α polymorph of indomethacin produced in the presence of poloxamer 407 (Figure 8A: IndAcP 20), a reduction in the onset temperature from 152.6 to 131.1 °C (a difference of over 20°) for the α peak was observed (versus Figure 8B: IndAc 6).

### 3.3 Molecular Modeling

To further study the influence of poloxamer 407 on the polymorphic outcome of indomethacin, we conducted molecular modeling. The influence of poloxamer 407 in the structure of the two polymorphs of indomethacin observed in the experiments was studied in detail. The α polymorphic form of indomethacin has three drug molecules in the asymmetric unit, with two molecules forming a mutually hydrogen-bonded carboxylic acid dimer, while the carboxylic acid of the third molecule is hydrogen bonded to one of the amide carbonyls of the dimer.^71^ In the γ polymorph of indomethacin, the only hydrogen bonds observed are two molecules forming a hydrogen-bonded carboxylic acid dimer.

**Figure 9:**
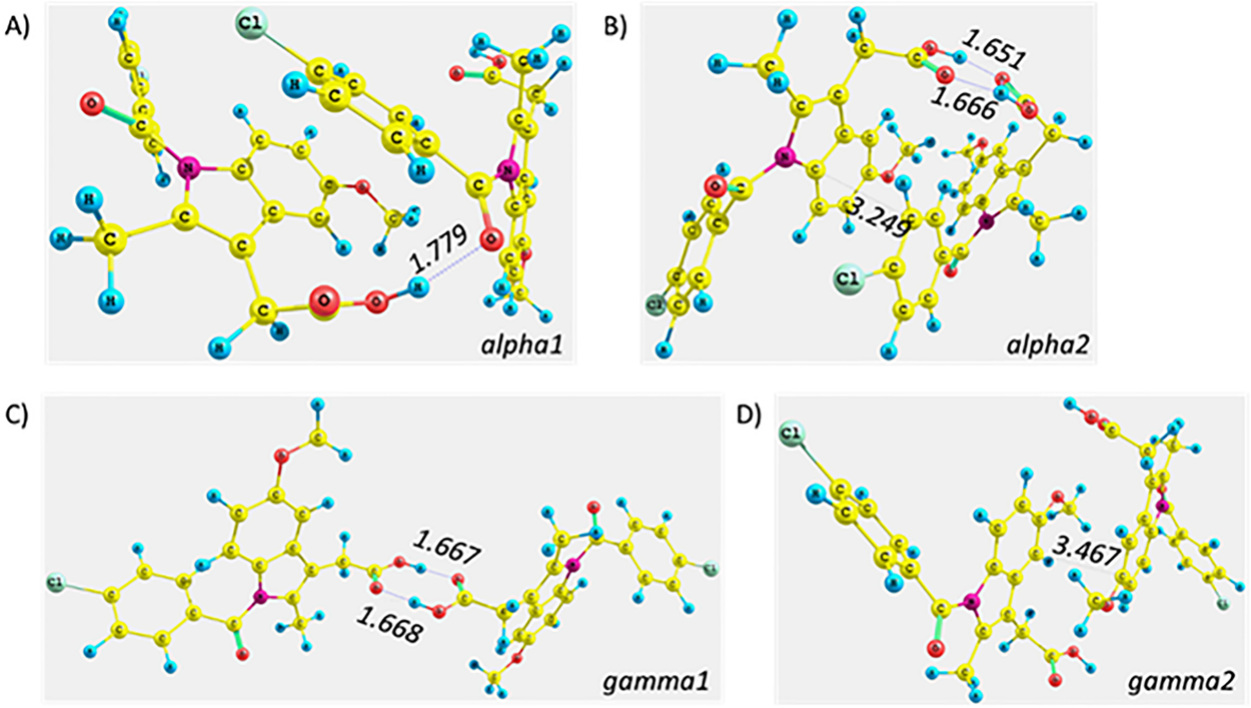
Optimized structure of (A) “alpha1” and (B) “alpha2” dimers of indomethacin corresponding to the α polymorph as well as (C) ”gamma1” and (D) “gamma2” dimers of indomethacin corresponding to the γ polymorph. The values presented are the distances of the bonds [Å].

First, indomethacin dimers were isolated from their crystal structures (α and γ polymorphs), two possible configurations (named 1 and 2) were optimized (Figure 9), and the interaction energy calculated the B97D3/6-311++G(d,p) level of theory. This interaction energy for the optimized “alpha1”, “alpha2”, “gamma1”, and “gamma2” dimers was −21.57, −35.77, −20.23, and −14.16 kcal/mol, respectively. Therefore, the most negative value of the interaction energy was calculated for the “alpha2” dimer. It could be due to the formation of two hydrogen bonds: C=O···H–O with a distance of 1.651 and 1.666 Å, and the close proximity of the phenyl rings (approximately 3.2 Å). The π–π (phenyl–phenyl) type of interaction was detected for the “gamma2” dimer (Figure 9D); however, additional hydrogen bonds were not formed.

The interactions within the above dimers were also assessed by the SAPT0 approach with the jun-cc-pVDZ basis set.^52^ The estimated values of the SAPT0 total energy were −22.58, −39.64, −23.43, and −15.21 kcal/mol for the A–D dimers (Figure 9), respectively. Taking into consideration the “alpha2” and “gamma2” dimers (Figure 9B, D), the major contribution to the total interaction energy was related to the electrostatic (−45.65 versus −10.59 kcal/mol for the “alpha2” or “gamma2” dimers, respectively) and dispersion (−28.52 versus −18.32 kcal/mol for the “alpha2” or “gamma2” dimers, respectively) energetic terms. These findings supported the conclusions drawn from the above interaction energy studies based on the BSSE factor, suggesting that the α polymorph of indomethacin was more energetically favorable as it is related to more negative values of the discussed energies.

In the next step, the interactions of the α and γ polymorphs of indomethacin with poloxamer were investigated. For this purpose, indomethacin molecules, as subunits (single molecules) extracted from the optimized “alpha2” or “gamma2” dimers, were docked to the polymer using the AutoDock Vina package,^57^ and the resulting configurations were subjected to semiempirical computations or taken for further molecular dynamics (MD) simulations. The estimated binding affinity values estimated from the docking procedure were −3.90 and −3.40 kcal/mol, for the “alpha2” or “gamma2” dimers, respectively, and suggested that the α polymorph of indomethacin might have a greater affinity to interact with poloxamer. However, the difference was not substantial, and thus MD simulations, which are more informative in nature, were conducted.

**Figure 10:**
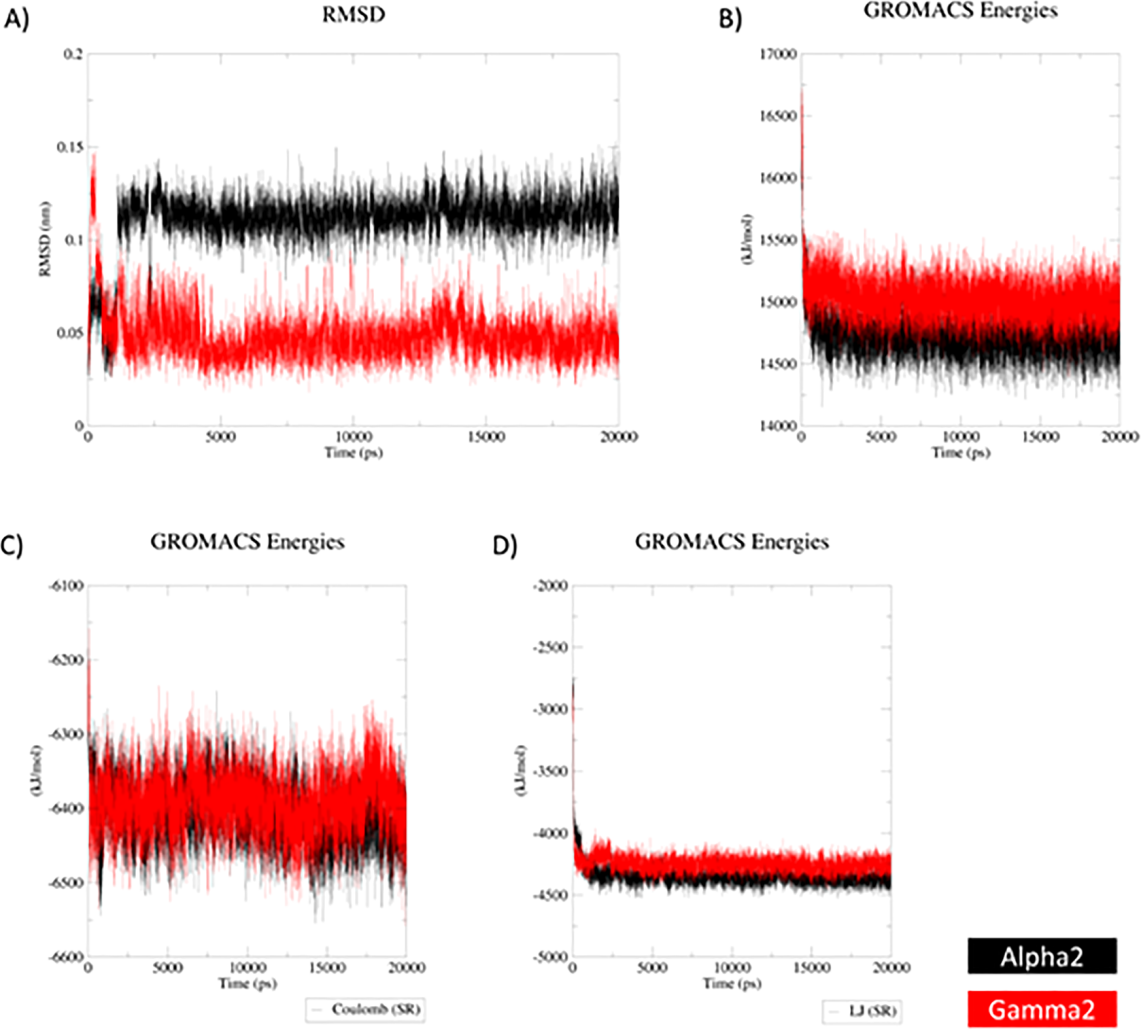
Energy plots representing the optimized “alpha2” (black) and “gamma2” (red) dimers. (A) RMSD plot for poloxamer in the ligand–polymer complex during the productive phase calculated for its complex with indomethacin dimers. (B) Total energy plot for poloxamer in the ligand–polymer complex during the productive phase calculated for its complex with indomethacin dimers. (C) Energy plot for Coulomb (electrostatic) interactions for the poloxamer within ligand–polymer complex during the productive phase calculated for its complex with indomethacin dimers. (D) Lennard–Jones potential plot for the poloxamer within ligand–polymer complex during the productive phase calculated for its complex with indomethacin dimers.

The ligand root-mean-square deviation (RMSD) plot (Figure 10A) showed that the docking configurations of all ligands inside the complex were stable. Generally, the ligands remained steady, as regards their impact on poloxamer, in their positions with an average RMSD of around 1.12 Å (“alpha2”) and 0.46 Å (“gamma2”). For the “alpha2” ligand, the initial RMSD was around 0.65 Å, and then at 1.84 ns, the RMSD curve moved upward and remained at 1.12 Å. In all cases, the RMSD values were smaller than 1.5 Å. The stability of indomethacin within the complex with poloxamer was shown by the analysis of the total energy during the simulations (Figure 10B) suggesting that the “alpha2” ligand was slightly more in comparison with “gamma2”. Although the electrostatic interactions within the analyzed complexes in the function of time remained similar (Figure 10C), it appeared that the van der Waals type of interactions presented in the plot of the Lennard–Jones plot (Figure 10D) seemed to be more important for the stability of the complex and showed a greater impact of the “alpha2” ligand on poloxamer as the average value was around 3496 and 3606 kJ/mol for the “alpha2” and “gamma2” ligands, respectively.

In the last step of the *in silico* experiments, the ability of the “alpha2” and “gamma2” ligands of indomethacin to interact with poloxamer was analyzed on the basis of semiempirical approach and the PM7 Hamiltonian as commonly used in the literature.^54−56,72^ The changes in enthalpy of indomethacin interactions in the complex with poloxamer was carried out using the geometries taken from the docking protocol. In this evaluation, the values of heat of formation (HOF) were considered under standard conditions using MOPAC2016 and its module, Mozyme.^53^ For the interaction energy calculations, an approach based on the thermodynamic cycle of Raha and Merz was adopted (eq 1^54^):
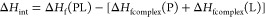
1where Δ*H*f(X) are the heats of formation *in vacuo* of the polymer–ligand complex, free ligand (L) or free polymer (P), and the Δ*H*_fcomplex_(X) parameter corresponds to the enthalpy of poloxamer or ligand molecule in the complex conformation. The application of the eq 1 to the complexes of ligands with poloxamer led to the values shown in Table 2. It can be concluded that the α polymorph of indomethacin appears to be more effective in the interaction with the poloxamer.

**Table 2:** Calculated Heats of Formation [kcal/mol] for Free Ligands (Δ*H*_fcomplex(L)_), Free Polymer (Δ*H*_fcomplex(P)_), Ligand–polymer Complex (Δ*H*_f(PL)_), As Well As Ligand–Polymer Interaction Energy (Δ*H*_int_)

indomethacin ligand	HOF of ligand (Δ*H*_fcomplex(L)_)	HOF of polymer (Δ*H*_fcomplex(P)_)	HOF of complex (Δ*H*_f(PL)_)	Δ*H*_int_
alpha2	–109.19	–6439.83	–6657.90	–108.89
gamma2	–149.03	–6495.93	–6733.75	–88.78

## 4 Conclusions

The polymorphic outcome of small-scale crystallisation events is challenging to control, as it was experimentally observed in the crystallization of indomethacin by gas antisolvent (GAS) without poloxamer 407. In the design of experiments (DoE) conducted without that additive, the γ and α polymorphs, a mix of both, or an acetone solvate was obtained. In the experiments by GAS where poloxamer 407 was used, the crystallization of indomethacin was steered toward the formation of the α polymorph of indomethacin. This fact indicates that there is a significant interaction between indomethacin and poloxamer 407. With molecular modeling, it was demonstrated that in the presence of poloxamer 407, the stability of a dimer from the α polymorph of indomethacin was superior to the dimers from the γ polymorph. Therefore, the GAS method together with the use of additives shows potential for controlling the polymorphism of APIs and contributes to the knowledge of the control of polymorphism of indomethacin using techniques based on supercritical CO_2_. Furthermore, it gives an insight and remarks on the importance of molecular modeling to understand the stabilization of binary systems composed of APIs and additives.

## Supporting Information Available

Schematic representation of the design of experiments (DoE) approach used for the gas antisolvent (GAS) process. This schematic representation is also presented in Figures 2, 3, 5 and 6, to describe the polymorphic outcome of indomethacin. Additional details on the DoE approach, including the mole fraction of CO_2_ and indomethacin, along with the duplicate experiments of the DoE approach (PDF)
