# Supplementary material for: Controlling the Polymorphism of Indomethacin with Poloxamer 407 in a Gas Antisolvent Crystallization Process
Source: ACS Omega. 2022 Nov 18;7(48):43945–57. doi: 10.1021/acsomega.2c05259 (PMC9730483; doi:10.1021/acsomega.2c05259)
Supplement: Supplementary file 1 — ao2c05259_si_001.pdf [file ao2c05259_si_001.pdf]

Supporting Information file

Controlling the polymorphism of indomethacin with poloxamer 407 in a gas antisolvent crystallisation process

Fidel Méndez Cañellas[1,2], Vivek Verma[1]**, Jacek Kujawski[3], Robert Geertman[4], Lidia Tajber[2,5], Luis Padrela[1,2]*

[1] Department of Chemical Sciences, Bernal Institute, University of Limerick, Limerick V94 T9PX, Ireland

[2] SSPC, the SFI Research Centre for Pharmaceuticals, Bernal Institute, University of Limerick, Limerick V94 T9PX, Ireland

[3] Chair and Department of Organic Chemistry, Faculty of Pharmacy, Poznan University of Medical Sciences, Grunwaldzka 6 street, 60-780 Poznan, Poland

[4] Janssen Pharmaceutica NV, 2340, Beerse, Belgium

[5] School of Pharmacy and Pharmaceutical Sciences, Trinity College Dublin, College Green, D02 PN40, Dublin 2, Ireland

* Corresponding author: Luis.Padrela@ul.ie

** Current address differs from the research affiliation. Vivek Verma is affiliated with Imperial College London, Department of Chemical Engineering, South Kensington Campus, SW7 2AZ, UK.

- Schematic representation of the DoE used for the Gas Antisolvent (GAS) process
- Additional details on the DoE
- Duplicates for the GAS experiments: Figures S2-S5

- **Schematic representation of the DoE used for the Gas Antisolvent (GAS) process**

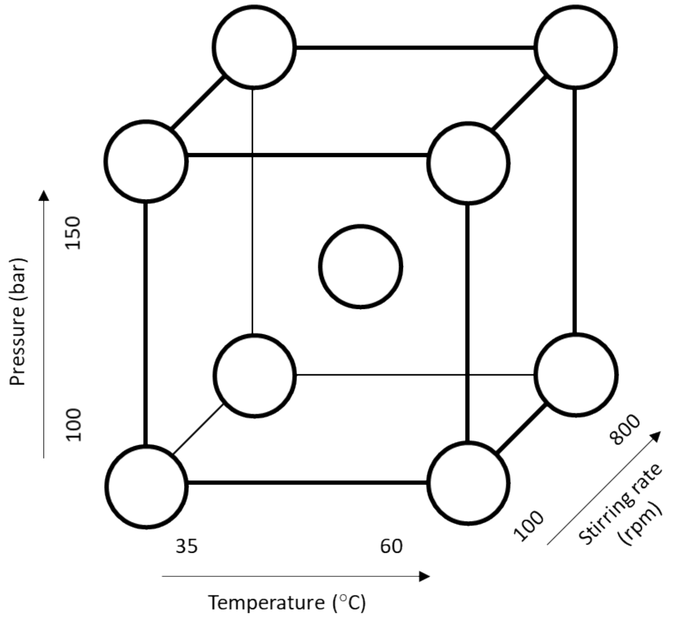

Figure S1. Design of Experiments (DoE) schematic to investigate the impact of pressure, temperature and stirring rate as process variables on the polymorphic outcome of indomethacin particles produced by the gas antisolvent (GAS) process.

- **Additional details on the DoE**

At a higher mole fraction of indomethacin, the supersaturation of the system was higher. It is important to remark that only the CO_2 mass varied throughout the experiments while the indomethacin and organic solvent mass remained constant, as the drug concentration in the initial solution is the same for all the experiments. At different values of pressure and temperature the CO_2 presents different densities and therefore, in the same volume of the high-pressure vessel it will present different mass ratios. In addition, local supersaturation may also vary according to the stirring rate. Nonetheless, a correlation between the CO_2 and indomethacin mole fractions present in each experiment and the polymorphic outcome of indomethacin could not be established.

Table S1. Mole fraction of CO_2 and indomethacin (Ind) in the gas antisolvent (GAS) experiments where no excipient was used. The mole fraction was calculated taking into consideration all the components in the high-pressure vessel during the precipitation. The density of the CO_2 at the determined conditions of temperature and pressure for the calculation of the mole fractions was extracted from the NIST Chemistry WebBook, SRD 69 (https://doi.org/10.18434/T4D303). The densities of acetone and ethyl acetate used for the calculations were 784 kg/m^3 and 902 kg/m^3 calculated at 25 °C as the solutions were sealed in the high-pressure vessel at room temperature.

Additive	Solvent	Pressure (MPa)	Temperature (°C)	Stirring rate (RPM)	Solid form obtained	Mole fraction	
						xCO_2	xInd
No additive	Acetone	10.0	35	100	γ /solvate	0.957813	0.000174
		15.0	35	100	α / solvate	0.962908	0.000153
		15.0	35	800	γ / α + γ	0.962908	0.000153
		10.0	35	800	γ / α + γ	0.957813	0.000174
		12.5	48	450	γ / α + γ	0.953233	0.000193
		10.0	60	100	α	0.902293	0.000403
		15.0	60	100	α	0.950594	0.000204
		15.0	60	800	α	0.950594	0.000204
		10.0	60	800	α / α + γ	0.902293	0.000403
	Ethyl acetate	10.0	35	100	α	0.967596	0.000176
		15.0	35	100	α + γ	0.971545	0.000154
		15.0	35	800	γ	0.971545	0.000154
		10.0	35	800	γ / α + γ	0.967596	0.000176
		12.5	48	450	α / α + γ	0.964039	0.000195
		10.0	60	100	α	0.923929	0.000413
		15.0	60	100	α	0.961985	0.000206
		15.0	60	800	α / α + γ	0.961985	0.000206
		10.0	60	800	α / γ	0.923929	0.000413

- **Duplicates for the GAS experiments: Figures S2-S5**

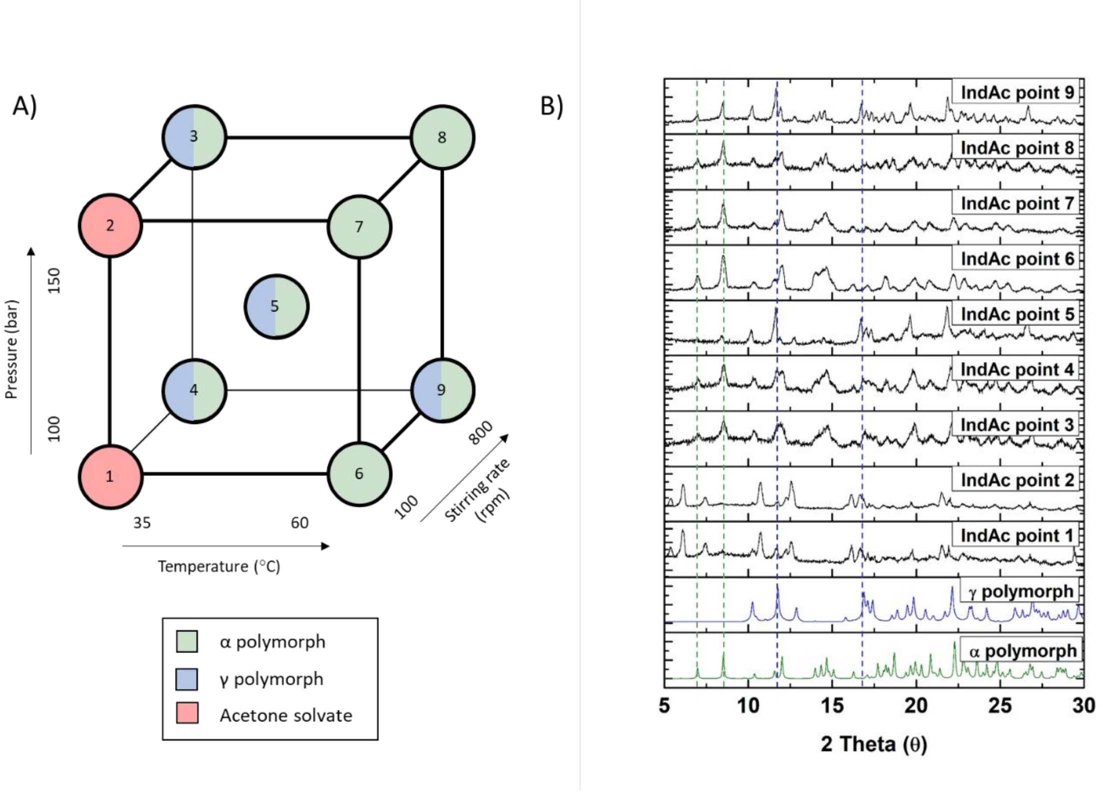

Figure S2. (A) Duplicate of the design of experiments (DoE) schematic to investigate the impact of pressure, temperature and stirring rate as the process variables on the polymorphic outcome of indomethacin particles produced by the gas antisolvent (GAS) process, using acetone as solvent. (B) X-ray powder diffraction (XRPD) patterns of the α and γ polymorphs of indomethacin from the Cambridge Structural Database (CSD) and DoE samples produced by the GAS method. Experimental conditions as described in Table 1 (DoE points IndAc 1-9). Green dotted lines indicate the characteristic peaks of the α polymorph at 7° and 8.5° 2θ, while the blue dotted lines indicate the characteristic peaks of the γ polymorph at 11.8° and 17° 2θ.

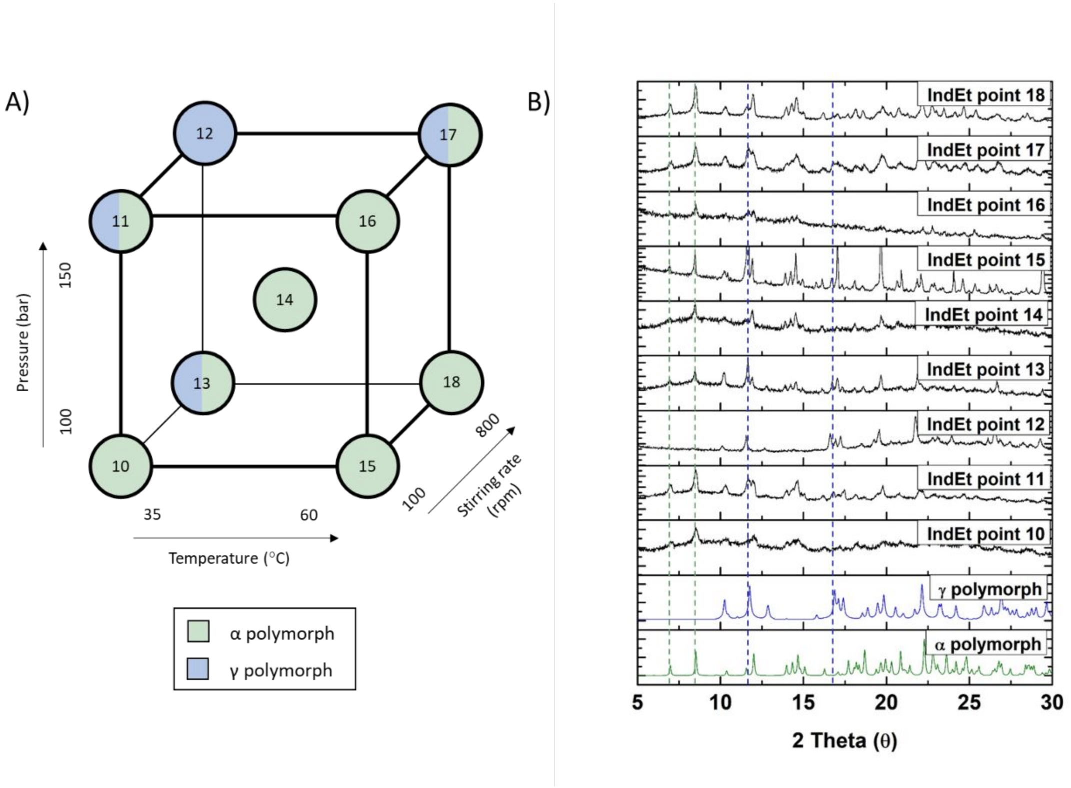

Figure S3 (A) Duplicate of the design of experiments (DoE) schematic to investigate the impact of pressure, temperature and stirring rate as the process variables on the polymorphic outcome of indomethacin particles produced by the gas antisolvent (GAS) process, using ethyl acetate as solvent. (B) X-ray powder diffraction (XRPD) patterns of the α and γ polymorphs of indomethacin from the Cambridge Structural Database (CSD) and DoE samples produced by the GAS method. Experimental conditions as described in Table 1 (DoE points IndEt 10-18). Green dotted lines indicate the characteristic peaks of the α polymorph at 7° and 8.5° 2θ, while the blue dotted lines indicate the characteristic peaks of the γ polymorph at 11.8° and 17° 2θ.

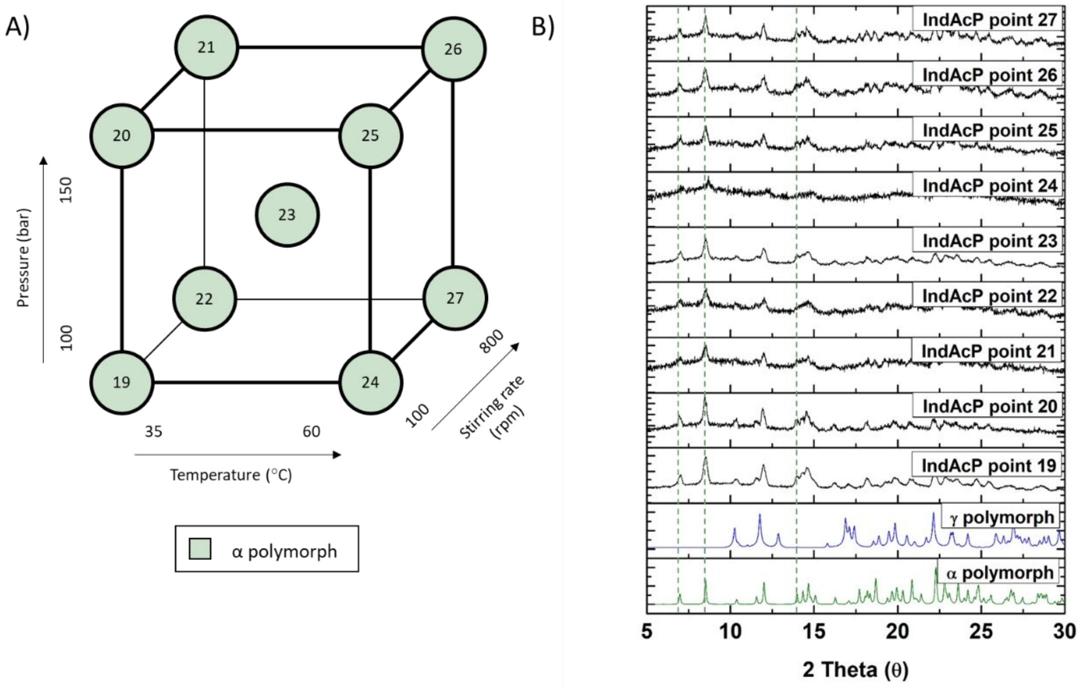

Figure S4. (A) Duplicate of the design of experiments (DoE) schematic to investigate the impact of pressure, temperature and stirring rate as the process variables on the polymorphic outcome of indomethacin particles produced by the gas antisolvent (GAS) process, using acetone as solvent and poloxamer 407 as additive. (B) X-ray powder diffraction (XRPD) patterns of the α and γ polymorphs of indomethacin from the Cambridge Structural Database (CSD) and DoE samples produced by the GAS method. Experimental conditions as described in Table 1 (DoE points IndAcP 19-27). Green dotted lines indicate the characteristic peaks of the α polymorph at 7°, 8.5° and 14.0° 2θ.

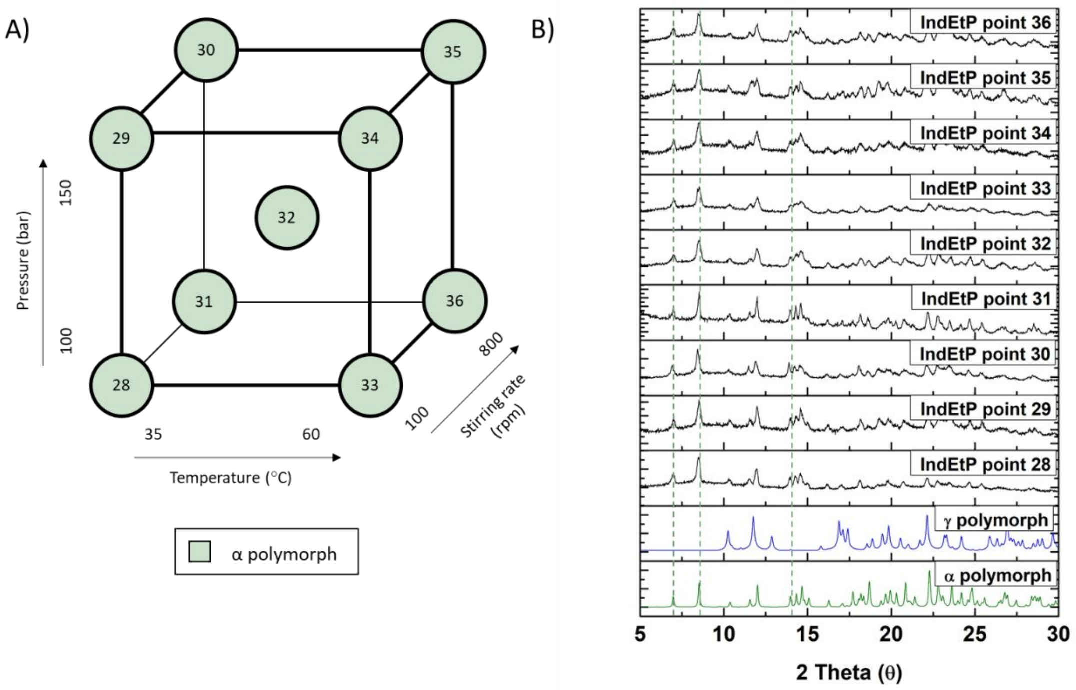

Figure S5. (A) Duplicate of the design of experiments (DoE) schematic to investigate the impact of pressure, temperature and stirring rate as the process variables on the polymorphic outcome of indomethacin particles produced by the gas antisolvent (GAS) process, using ethyl acetate as solvent and poloxamer 407 as additive. (B) X-ray powder diffraction (XRPD) patterns of the α and γ polymorphs of indomethacin from the Cambridge Structural Database (CSD) and DoE samples produced by the GAS method. Experimental conditions as described in Table 1 (DoE points IndEtP 28-36). Green dotted lines indicate the characteristic peaks of the α polymorph at 7°, 8.5° and 14.0° 2θ.
